# Using Convolutional Neural Networks to Efficiently Extract Immense Phenological Data From Community Science Images

**DOI:** 10.3389/fpls.2021.787407

**Published:** 2022-01-17

**Authors:** Rachel A. Reeb, Naeem Aziz, Samuel M. Lapp, Justin Kitzes, J. Mason Heberling, Sara E. Kuebbing

**Affiliations:** ^1^Department of Biological Sciences, University of Pittsburgh, Pittsburgh, PA, United States; ^2^Section of Botany, Carnegie Museum of Natural History, Pittsburgh, PA, United States

**Keywords:** phenology, deep learning, citizen science, iNaturalist, *Alliaria petiolata* (garlic mustard), convolutional neural network

## Abstract

Community science image libraries offer a massive, but largely untapped, source of observational data for phenological research. The iNaturalist platform offers a particularly rich archive, containing more than 49 million verifiable, georeferenced, open access images, encompassing seven continents and over 278,000 species. A critical limitation preventing scientists from taking full advantage of this rich data source is labor. Each image must be manually inspected and categorized by phenophase, which is both time-intensive and costly. Consequently, researchers may only be able to use a subset of the total number of images available in the database. While iNaturalist has the potential to yield enough data for high-resolution and spatially extensive studies, it requires more efficient tools for phenological data extraction. A promising solution is automation of the image annotation process using deep learning. Recent innovations in deep learning have made these open-source tools accessible to a general research audience. However, it is unknown whether deep learning tools can accurately and efficiently annotate phenophases in community science images. Here, we train a convolutional neural network (CNN) to annotate images of *Alliaria petiolata* into distinct phenophases from iNaturalist and compare the performance of the model with non-expert human annotators. We demonstrate that researchers can successfully employ deep learning techniques to extract phenological information from community science images. A CNN classified two-stage phenology (flowering and non-flowering) with 95.9% accuracy and classified four-stage phenology (vegetative, budding, flowering, and fruiting) with 86.4% accuracy. The overall accuracy of the CNN did not differ from humans (*p* = 0.383), although performance varied across phenophases. We found that a primary challenge of using deep learning for image annotation was not related to the model itself, but instead in the quality of the community science images. Up to 4% of *A. petiolata* images in iNaturalist were taken from an improper distance, were physically manipulated, or were digitally altered, which limited both human and machine annotators in accurately classifying phenology. Thus, we provide a list of photography guidelines that could be included in community science platforms to inform community scientists in the best practices for creating images that facilitate phenological analysis.

## Introduction

The study of phenology, or the timing of life cycle events, provides researchers with key insights into the role of time, as an axis, in ecological communities. Studies that monitor shifts in phenology are important for predicting the effects of environmental drivers, such as climate change, on species’ fitness, ecological interactions, ecosystem processes, and evolution (Forrest and Miller-Rushing, 2010). New frontiers in phenological research seek to assess the effects of large-scale environmental drivers on phenology and must be able to evaluate across multiple temporal and spatial scales (Cleland et al., 2007; Gallinat et al., 2021). This endeavor requires access to sources of phenological data that are both temporally and spatially extensive. However, owing to the high data requirement of phenological studies, previous researchers have been limited in their ability to assess phenological questions which are spatially and temporally explicit in tandem (Wolkovich et al., 2014). Studies that assess a large temporal period of phenology, such as those that utilize historic herbarium records of phenology, are typically spatially limited to the local-scale or to a defined number of sites across a larger region (Primack et al., 2004; Cook et al., 2012; Hart et al., 2014; Park and Schwartz, 2015; Reeb et al., 2020). By contrast, studies that assess a large spatial area (such as at continental scales), are typically restricted to a short frame in time or phenological observations are aggregated across years (Li et al., 2019, but see Templ et al., 2018).

Up to this point, such sources of phenological data have not been easily available (Tang et al., 2016). However, community science platforms designed to document species diversity, such as iNaturalist, or eBird, provide a rich source of spatially and temporally extensive phenology data (Sullivan et al., 2009; Barve et al., 2020; Li et al., 2021). In 2020 alone, iNaturalist users logged 12.6 million research-grade observations and eBird users logged 169 million observations (eBird, 2021; iNaturalist, 2021)^1,2^. However, because these biodiversity-focused community science programs are not designed to track species’ phenology, images in biodiversity-focused databases must be manually annotated for phenology. Though images from community science platforms have been powerfully leveraged in phenology research (Barve et al., 2020; Li et al., 2021), it requires much effort to visually score many thousands of images. Currently, the most common practice for rapidly annotating large numbers of images is by employing non-expert scorers, such as undergraduate students and volunteers, or through crowdsourcing platforms like Mechanical Turk (Willis et al., 2017). Not all researchers are able to utilize this method, as it can be costly or require access to a skilled labor pool (McDonough MacKenzie et al., 2020). The effort and costs associated with sorting and categorizing images into phenological stages means these community science biodiversity databases are underutilized in phenological research. A low-cost, precise, and efficient method for categorizing images in community science biodiversity databases would complement data available in other community science datasets that are specifically generated for phenological research, such as the National Phenology Network’s Nature’s Notebook, which are more limited in their spatial coverage (2.8 million observations in 2020) (Crimmins, 2021), as well as initiatives combining disparate phenological datasets (e.g., Brenskelle et al., 2019)^3^.

A promising solution to accessing the copious phenological data embedded in community science biodiversity datasets lies in automated image classification. Convolutional neural networks (CNNs) are a widely used machine learning technique for image classification (Affonso et al., 2017; Wäldchen and Mäder, 2018; Christin et al., 2019). These neural networks extract important features like lines, shapes, and colors and uses these features to classify an image into pre-designated categories (Rawat and Wang, 2017). Researchers can train CNNs to classify novel image sets by providing a training data set of pre-classified images. The CNN network then learns by iteratively predicting the classification labels of samples, comparing its classification labels to the true labels, and updating parameters within the network accordingly (Rawat and Wang, 2017). Once trained, CNNs can classify novel images rapidly and accurately. Researchers have employed CNNs to classify large image datasets including identifying animal species in wildlife camera trapping images (Tabak et al., 2019) or quantifying herbivory damage and leaf area on herbarium specimens (Meineke et al., 2020; Weaver et al., 2020).

Recently, immense progress has been made in applying deep learning models to phenological studies using herbarium specimens (Pearson et al., 2020) and aerial images (Pearse et al., 2021). Pearse et al. (2021) utilized a CNN approach to classify tree species from aerial images and found that tree phenology substantially influenced model accuracy. Lorieul et al. (2019) utilized a CNN approach to classify images of herbarium specimens into specific phenophases, while Davis et al. (2020) and Goëau et al. (2020) utilized a mask R-CNN approach to detect and count the number of reproductive structures on herbarium specimens. In these applications, researchers demonstrated that deep learning models are highly useful and with accuracy rates that rival manual (human) annotation (Lorieul et al., 2019). However, the classification of plant phenology in community science images presents a new challenge for CNNs. Unlike images of herbarium records that are mounted and photographed in a standardized fashion, images of plants in the field vary widely in the background environment, the distance of a plant to the camera, the resolution of an image, and light conditions (Barve et al., 2020). Increased image variability could reduce the accuracy, and thus utility, of using deep neural networks to classify plant phenology. Additionally, high image variability might inflate the required size of the training dataset to an unreasonably large number of images (Willi et al., 2019). Thus, it remains to be seen what the true threshold of neural network performance is when annotating phenology in community science images.

Here, we evaluate the potential use of deep neural networks to automatically classify the phenology of community science images uploaded to the iNaturalist platform. We focus on *Alliaria petiolata* (Brassicaceae; garlic mustard), a biennial herb that is common in Europe, western Asia, and widely naturalized in forests of eastern North America. In its first year, *A. petiolata* is a low-growing rosette of leaves and reproduces in mid-spring of its second year (Anderson et al., 1996). We selected *A. petiolata* because it is the fourth-most observed plant species on iNaturalist (over 40,000 research-grade observations between 1995 and 2020) and has distinct reproductive structures that can be identified from images. We ask the following questions: (1) How effective are CNNs in identifying phenology in a 2-phase and a 4-phase classification scheme? (2) How does CNN performance compare against the current best-practice, non-expert human scoring? Finally, based upon CNN performance, we recommend “best practices” for community scientists uploading images into community science platforms to enhance future phenology research.

## Materials and Methods

### Creating a Training and Validation Image Set

We downloaded 40,761 research-grade observations of *A. petiolata* from iNaturalist, ranging from 1995 to 2020. Observations on the iNaturalist platform are considered “research-grade if the observation is verifiable (includes image), includes the date and location observed, is growing wild (i.e., not cultivated), and at least two-thirds of community users agree on the species identification. From this dataset, we used a subset of images for model training. The total number of observations in the iNaturalist dataset are heavily skewed toward more recent years. Less than 5% of the images we downloaded (*n* = 1,790) were uploaded between 1995 and 2016, while over 50% of the images were uploaded in 2020. To mitigate temporal bias, we used all available images between the years 1995 and 2016 and we randomly selected images uploaded between 2017 and 2020. We restricted the number of randomly selected images in 2020 by capping the number of 2020 images to approximately the number of 2019 observations in the training set. The annotated observation records are available in the Supplementary Data Sheet 1. The majority of the unprocessed records (those which hold a CC-BY-NC license) are also available on GBIF.org (2021).

One of us (RR) annotated the phenology of training and validation set images using two different classification schemes: two-stage (non-flowering, flowering) and four-stage (vegetative, budding, flowering, and fruiting). For the two-stage scheme, we classified 12,277 images and designated images as “flowering” if there was one or more open flowers on the plant. All other images were classified as non-flowering. For the four-stage scheme, we classified 12,758 images. We classified images as “vegetative” if no reproductive parts were present, “budding” if one or more unopened flower buds were present, “flowering” if at least one opened flower was present, and “fruiting” if at least one fully-formed fruit was present (with no remaining flower petals attached at the base). Phenology categories were discrete; if there was more than one type of reproductive organ on the plant, the image was labeled based on the latest phenophase (e.g., if both flowers and fruits were present, the image was classified as fruiting).

For both classification schemes, we only included images in the model training and validation dataset if the image contained one or more plants with clearly visible reproductive parts were clear and we could exclude the possibility of a later phenophase. We removed 1.6% of images from the two-stage dataset that did not meet this requirement, leaving us with a total of 12,077 images, and 4.0% of the images from the four-stage leaving us with a total of 12,237 images. We then split the two-stage and four-stage datasets into a model training dataset (80% of each dataset) and a validation dataset (20% of each dataset).

### Training a Two-Stage and Four-Stage Convolutional Neural Network

We adapted techniques from studies applying machine learning to herbarium specimens for use with community science images (Lorieul et al., 2019; Pearson et al., 2020). We used transfer learning to speed up training of the model and reduce the size requirements for our labeled dataset. This approach uses a model that has been pre-trained using a large dataset and so is already competent at basic tasks such as detecting lines and shapes in images. We trained a neural network (ResNet-18) using the PyTorch machine learning library (Paszke et al., 2019) within Python. We chose the ResNet-18 neural network because it had fewer convolutional layers and thus was less computationally intensive than pre-trained neural networks with more layers. In early testing we reached desired accuracy with the two-stage model using ResNet-18. ResNet-18 was pre-trained using the ImageNet dataset, which has 1,281,167 images for training (Deng et al., 2009). We utilized default parameters for batch size (4), learning rate (0.001), optimizer (stochastic gradient descent), and loss function (cross entropy loss). Because this led to satisfactory performance, we did not further investigate hyperparameters.

Because the ImageNet dataset has 1,000 classes while our data was labeled with either 2 or 4 classes, we replaced the final fully-connected layer of the ResNet-18 architecture with fully-connected layers containing an output size of 2 for the 2-class problem and 4 for the 4-class problem. We resized and cropped the images to fit ResNet’s input size of 224 × 224 pixels and normalized the distribution of the RGB values in each image to a mean of zero and a standard deviation of one, to simplify model calculations. During training, the CNN makes predictions on the labeled data from the training set and calculates a loss parameter that quantifies the model’s inaccuracy. The slope of the loss in relation to model parameters is found and then the model parameters are updated to minimize the loss value. After this training step, model performance is estimated by making predictions on the validation dataset. The model is not updated during this process, so that the validation data remains “unseen” by the model (Alexander et al., 1995; Rawat and Wang, 2017). This cycle is repeated until the desired level of accuracy is reached. We trained our model for 25 of these cycles, or epochs. We stopped training at 25 epochs to prevent overfitting, where the model becomes trained too specifically for the training images and begins to lose accuracy on images in the validation dataset (Alexander et al., 1995).

We evaluated model accuracy and created confusion matrices using the model’s predictions on the labeled validation data. This allowed us to evaluate the model’s accuracy and which specific categories are the most difficult for the model to distinguish. For using the model to make phenology predictions on the full, 40,761 image dataset, we created a custom dataloader function in PyTorch using the Custom Dataset function, which would allow for loading images listed in a csv and passing them through the model associated with unique image IDs.

### Hardware Information

Model training was conducted using a personal laptop (Ryzen 5 3500U cpu and 8 GB of memory) and a desktop computer (Ryzen 5 3600 cpu, NVIDIA RTX 3070 GPU and 16 GB of memory).

### Comparing Convolutional Neural Network Accuracy to Human Annotation Accuracy

We compared the accuracy of the trained CNN to the accuracy of seven inexperienced human scorers annotating a random subsample of 250 images from the full, 40,761 image dataset. An expert annotator (RR, who has over a year’s experience in annotating *A. petiolata* phenology) first classified the subsample images using the four-stage phenology classification scheme (vegetative, budding, flowering, and fruiting). Nine images could not be classified for phenology and were removed. Next, seven non-expert annotators classified the 241 subsample images using an identical protocol. This group represented a variety of different levels of familiarity with *A. petiolata* phenology, ranging from no research experience to extensive research experience (two or more years working with this species). However, no one in the group had substantial experience classifying community science images and all were naïve to the four-stage phenology scoring protocol. The trained CNN was also used to classify the subsample images. We compared human annotation accuracy in each phenophase to the accuracy of the CNN using students *t*-tests. The model and human annotated subsample data can be found in the Supplementary Data Sheet 2. This research is exempt from University of Pittsburgh IRB approval according to the University’s Exempt Criteria 45 CFR 46.104(d)(2).

### Unclassifiable Images

Within the four-stage training and validation dataset, we removed 4% of plant images that could not be classified into a phenological stage. To quantitatively assess the cause of unclassifiable images, the experienced annotator (RR) labeled these images in one of six categories: (1) camera distance (camera was too far or too close to the plant to classify phenology), (2) physical manipulation (the plant was no longer rooted in the ground), (3) digital manipulation (the image was digitally altered or was copied from a secondary source), (4) senesced plant (no remaining leaves), (5) misidentified species (image did not contain *A. petiolata)*, and 6) duplicate entry (an image had been logged two or more times by the same user).

## Results

### Accuracy of a Two-Stage and Four-Stage Convolutional Neural Network

**FIGURE 1:**
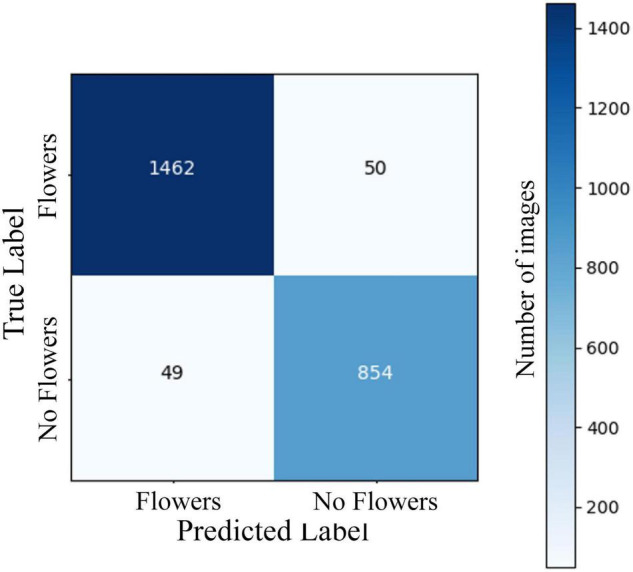
A confusion matrix showing model predictions vs. expert assigned labels for a CNN predicting 2-stage phenology of *A. petiolata* in a validation set of 2,415 iNaturalist images. Rows represent true labels of images, assigned by an expert annotater, columns represent label assigned by the CNN, and the numbers in cells represent the number of images within each category. Cells on the diagonal from the top left to bottom right represent correct model classifications. Overall CNN accuracy was 95.9%. The CNN was constructed using ResNet 18 and trained on 9,662 images of *A. petiolata* from iNaturalist.

**FIGURE 2:**
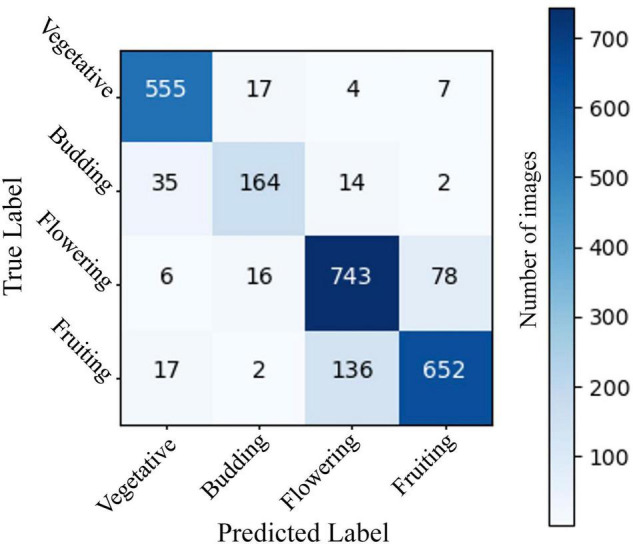
A confusion matrix showing model predictions vs. expert assigned labels for a CNN predicting 4-stage phenology of *A. petiolata* in a validation set of 2,448 iNaturalist images. Rows represent true labels of images, assigned by an expert scorer, columns represent label assigned by the CNN, and the numbers in cells represent the number of images within each category. Cells on the diagonal from the top left to bottom right represent correct CNN classifications. Overall CNN accuracy was 86.3%. The neural network was constructed using ResNet 18 and trained on 9,789 test images of *A. petiolata* from iNaturalist.

The two-stage CNN we trained to identify flowering vs. non-flowering images was able to correctly categorize 95.9% of images in a 2,415-image test dataset (Figure 1). The four-stage CNN we trained to identify vegetative, budding, flowering, and fruiting images was able to identify 86.4% of images from a 2,448-image test dataset (Figure 2). The drop in accuracy from the two-stage CNN is largely attributable to confusion between flowering and fruiting images. The four-stage CNN incorrectly classified 131 fruiting images as flowering, and 81 flowering images as fruiting. Confusion of a flowering plant for a fruiting plant or vice versa accounted for 64% of incorrectly classified images. All other mistaken classification happened much less frequently, the next most common mistake being 33 budding plants classified as vegetative.

### Comparing Convolutional Neural Network Accuracy to Human Annotation Accuracy

**FIGURE 3:**
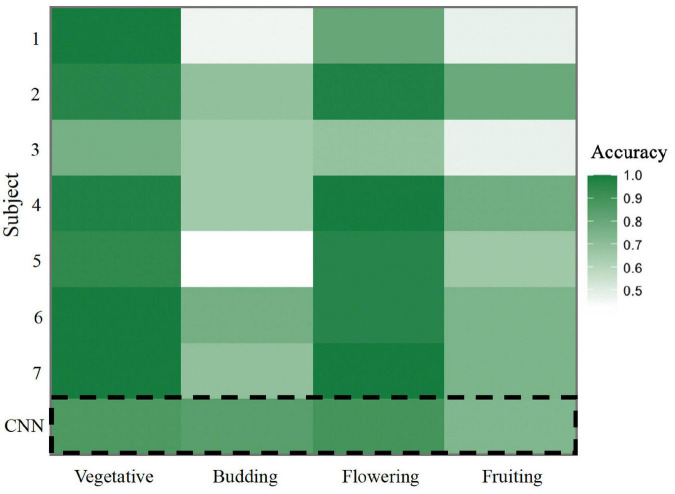
Heat map comparing non-expert human accuracy to CNN accuracy in annotating a dataset of 241 *A. petiolata* iNaturalist images. Accuracy was calculated as the percent of correctly annotated images in each phenophase. Each row represents the accuracy of a non-expert individual, with the exception of the bottom row (dashed line box) which represents the accuracy of the CNN.

To evaluate the usefulness of image classification by CNNs, we compared the accuracy of the trained four-stage CNN in classifying a random subsample of 241 images from the full dataset to a group of seven non-expert human annotators. Overall, the accuracy of the CNN did not differ significantly from humans (*p* = 0.383; Figure 3). The CNN correctly classified 81.7% of images while the non-expert group correctly classified 78.6% of images on average, with individual non-expert accuracy ranging from 60.9 to 86.6%. Evaluating individual phenophases, we found that the CNN was marginally less accurate than humans at identifying vegetative images (8% higher human accuracy, *p* = 0.053), but significantly more accurate in identifying budding images (23% lower human accuracy, *p* = 0.003). For flowering and fruiting phenophases, we found no difference in accuracy between the CNN and humans (*p* = 0.89 and *p* = 0.23, respectively).

### Fully Annotated Dataset

**FIGURE 4:**
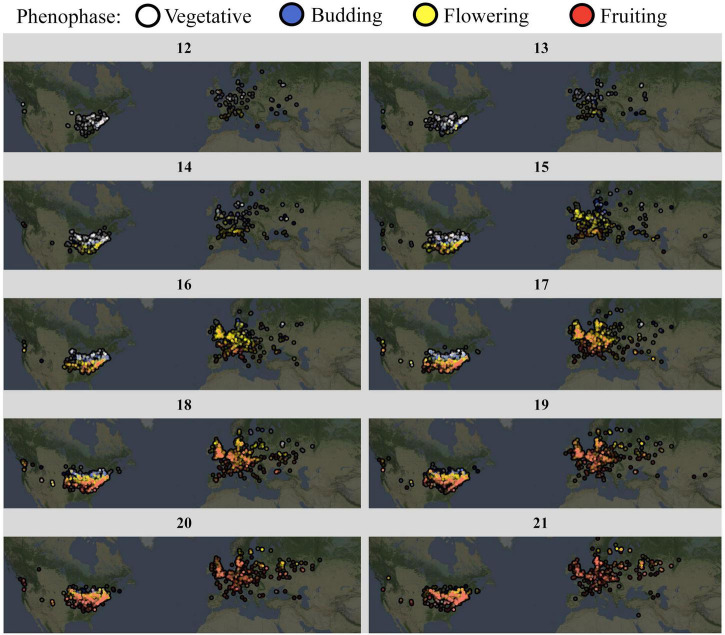
*A. petiolata* phenology progression, by calendar week. Weekly maps depict *A. petiolata* phenology using the full, CNN-annotated iNaturalist image set. Date ranges from early March (week 12) to mid-may (week 21). Observations have been combined across years (1995–2020). Color depicts phenophase: vegetative observations are white, budding observations are blue, flowering observations are yellow, and fruiting observations are red.

We finally used the trained CNN to annotate the full *A. petiolata* dataset, which contained 40,761 images. This set of community science images represented observations spanning the entire species range, 48 countries, and 15 years (Figure 4). The vast majority of observations (>80%) were recorded between the months of March and June, encapsulating the reproductive season for this species (Figure 4). The CNN classified 24.5% of images as vegetative, 9.3% as budding, 34.0% as flowering, and 32.2% as fruiting.

### Unclassifiable Images

Among unclassifiable images that were removed from the 4-stage training and validation dataset, the majority were removed owing to issues with the image quality for phenology use. We removed 57.1% of the images (*n* = 366) because the camera was too close or too far from the plant to determine the plant’s phenology, 21.2% (*n* = 136) because the photo captured a physically manipulated plant, and 1.4% (*n* = 9) because the photographer digital alterated the image. A minority of images were removed owing to other reasons. We removed 15.6% (*n* = 100) of images because the plant was senesced, 4.1% (*n* = 26) because the image did not contain the correct species, and 0.6% (*n* = 4) because the image was a duplicate entry.

The proportion of “unclassifiable” images has roughly declined over time (Supplementary Figure 1), signaling an overall improvement in image quality over time. Within the expert-annotated training image set, the annual proportion of unclassifiable images was 21% in 2010, 6% in 2015, and 4% in 2020.

## Discussion

Community science biodiversity databases are rich with observations of species phenology. However, a researchers’ ability to use this phenological information is limited because it is contained within images that require manual classification, which is a time-consuming and costly endeavor. Here, we determine the effectiveness of CNNs to automatically classify the phenology of a widespread plant, *Alliaria petiolata* (garlic mustard), using images uploaded to the community science platform iNaturalist. We find that CNNs are efficient and effective at classifying images into both a coarse two-phase classification scheme and a finer four-phase classification scheme. We also demonstrate that the CNN performed similarly to annotation by non-experts, which is currently the most popular method for large-scale phenology annotation. Thus, we conclude that CNNs, once trained, hold immense potential to serve as an inexpensive, rapid method of phenological data extraction from large community science image databases like iNaturalist.

This neural network does not require an extensive background in deep learning techniques to be utilized by researchers. It utilizes an open-source CNN (ResNet-18) that is pre-trained to identify images into 1000 object categories. By using a pre-trained model, we reduced the computing power necessary for training the algorithm to classify images of plants into specific phenophases. By using open-source machine learning functions from the PyTorch libraries in Python (Paszke et al., 2019), we also reduced our coding time. PyTorch is an open-source machine learning library, which provides functions for model-building and evaluation as well as an extensive array of tutorials for learning (Pytorch, 2021)^4^. Thus, the CNN presented in this project could be re-trained to classify phenology for another set of images, even for researchers with limited deep learning knowledge or computing resources. Based on our informal observations during model development, we found that a coarse and simple two-stage phenology classifier (such as flowering/non-flowering) can be trained using as few as 2,000 images and, in our experience, requires the computing power of a modern personal laptop. Our four-stage phenology classifier required a significantly larger training set of 12,758 images to reach the existing level of performance (86.4% accuracy). We did not evaluate whether the performance of the four-stage classifier would improve with a larger training and validation sets. In our experience, training a model of this size can be accomplished on a standard modern desktop. The usefulness of CNNs for phenological data extraction will depend on the phenology classification scheme and the number of images in the dataset. We were able to extract four-stage phenological information from the full, 40,761 image set of *A. petiolata* using a model trained off a subsample of 12,758 images; reducing the time for human annotation by 68.7%. Researchers who wish to utilize a finer-scale phenology classification scheme (ex 6-stage) will likely require a larger training image set.

**FIGURE 5:**
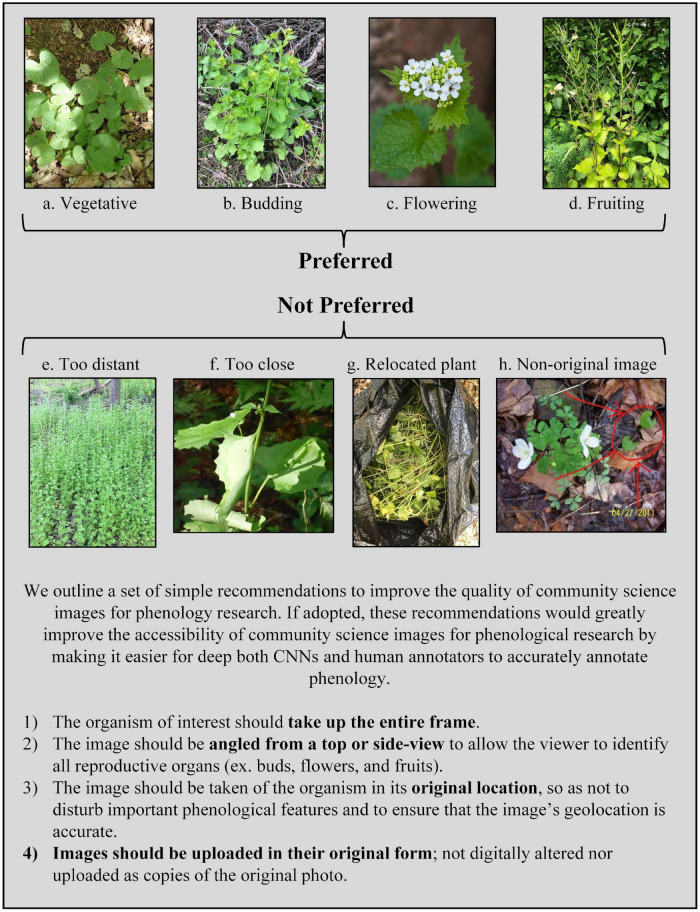
Description of preferred vs. non-preferred community science images for use in phenology research. Example images were sourced from iNaturalist. Image credit (iNaturalist username): **(a)** szuwarek; **(b)** deannahunt; **(c)** midnight_jim; **(d)** pedropedreiro; **(e)** pleasethetrees; **(f)** ny_wetlander; **(g)** r_rogge; **(h)** chuckt2007.

The CNN’s accuracy in classifying images hinged on the quality of the images, not the quality of the model. While some failures in image quality are unavoidable, such as a mistaken species identity or images taken of senesced plants, the majority of failures can be attributed to avoidable photographer choices. We found that iNaturalist images were highly variable in the following three aspects: the distance between the photographer and the plant, the level of physical manipulation of the plant, and the degree of digital alteration of the photograph (Figure 5). The distance from the camera to the plant varied from a few decimeters (for example, close-up pictures of a single flower) to several meters. At both ends of this range, a plant’s phenology can be misidentified. For example, when photographers upload close-up images of a single flower, other reproductive organs may be left out of the image frame. If a plant is also developing fruits, but those reproductive organs are not captured in an image of flowers, the image may be incorrectly classified as “flowering” instead of “fruiting.” Likewise, when photographers upload images of a plant from too far away, the reproductive organs may be too small or too blurry to accurately classify. Some photographers remove plants and move them to a new location (such as indoors) or press them for herbarium records. When photographers move or manipulate plants, reproductive organs can fall off the plant or be missed in the image, reducing the CNN’s accuracy of image classification. Lastly, some photographers will upload non-original images that are either digitally altered or are copies of pre-existing images. This can lead to distortion of the image and difficulty in identifying reproductive organs. Researchers are also unable to trust the geospatial information associated with these images because there is no way to verify where the coordinates were generated.

We were unable to manually classify the four-stage phenology of 4.0% of *A. petiolata* images because they did not contain the key features required to classify phenology. While expert human annotators can manually remove unclassifiable images from analysis, our trained CNN was forced to label them into a pre-defined category. This ultimately increases the number of incorrect classifications within the annotated dataset when using deep neural networks. However, as long as the occurrence rate of unclassifiable images is small, it is unlikely to introduce substantial error within large datasets. The higher quality the community science image, the greater the ability for the model to annotate phenology accurately without encountering unclassifiable images. In our analysis, we found that the percentage of unclassifiable images has roughly decreased over time, having declined from 21% of images in 2010 to just 4% of images in 2020. We expect image quality to continue to improve, owing to the widespread use of smartphones with ever-increasing associated camera capabilities and an increasing public awareness of proper photography techniques.

We outline a set of simple recommendations to improve the quality of community science images for phenology research (Figure 5). If adopted by community photographers, these recommendations would greatly improve the accessibility of community science images for phenological research by making it easier for both deep neural networks and human annotators alike to accurately annotate phenology. We hope that these will be useful to community science platforms for informing their educational material, or directly useful to community scientists who wish to individually contribute their images to research. First, the organism of interest should take up the entire frame. Second, the image should be angled to allow the viewer to identify all reproductive organs (ex. buds, flowers, and fruits). Third, the image should be taken of the organism in its original location, so as not to disturb important phenological features and to ensure that the image’s geolocation is accurate. Fourth, images should be uploaded in their original form; not digitally altered nor uploaded as copies of the original photo. Ideally, additional images are uploaded to show additional features of interest, including detailed characters needed for identification or otherwise worth documenting, as well as images showing the population, community, and environmental context of the individual plant. However, the first picture uploaded should capture the standardized recommendations we suggest to specifically capture the individual at the whole organism level.

Importantly, we found that a CNN performed similarly to non-expert human annotators in their phenology classification accuracy. Human annotators had the lowest accuracy in the budding and fruiting phases (61.8 and 67.1%, respectively). While the bright white flowers of *A. petiolata* are easy to identify, the green buds and fruits are substantially easier to miss without a trained eye, leading to an increase in error for inexperienced human annotators. The trained CNN had equal difficulty in identifying fruits but was substantially better at identifying budding images than human annotators. Budding images are uncommon among *A. petiolata* images (roughly 10%). We predict that human annotators had more difficulty in identifying buds due to an unconscious cognitive bias in searching for common features and overlooking rare ones. The CNN may have been less susceptible to bias introduced by an underrepresented class such as budding phase *A. petiolata*; suggesting an advantage to using this tool.

Furthermore, CNNs can be a substantially faster and less-expensive method of data collection when compared to inexperienced human annotators. In general, we found that inexperienced humans could annotate 2–5 images per minute. This would translate to at least 135 h of paid labor if a person were to manually annotate the full 40,761 image dataset of *A. petiolata*. By contrast, the CNN annotated the full image dataset in under 2 h. After the initial time investment required to annotate a training dataset and write the code for a CNN, images can be annotated more rapidly using CNNs than with manual annotation. While the initial time investment in the development of a machine learning model is steep, the speed at which a CNN can annotate a large dataset produces exponential gains in efficiency as dataset size increases.

It will be imperative for CNN-generated phenological data to align with standardized phenological ontologies like the standardized herbarium specimen digitization protocol developed by Yost et al. (2018) and the Plant Phenology Ontology developed by Stucky et al. (2018). This alignment would allow researchers to compare iNaturalist, herbarium, or *in situ* phenology data and promote the inclusion of iNaturalist phenological data in larger compiled databases. For example, the two-stage phenology scheme used in this study provides a coarse level of phenological information, akin to first-order scoring as described in Yost et al. (2018). The four-stage scheme provides a finer scale of phenological information, akin to second-order scoring (Yost et al., 2018). The integration of standardized ontologies will allow CNN-generated data to be ingested into larger phenology databases such as plantphenology.org (Brenskelle et al., 2019), enabling future data reuse and interoperability.

We successfully trained a CNN to extract phenological data from community science images of *A. petiolata*. We note that we intentionally selected *A. petiolata* because its reproductive organs are visible from a distance (and can be identified in photos) and it was abundant within the iNaturalist database. For researchers who wish to study species that have lower availability of images in community science databases or hard-to-distinguish reproductive organs, they may need to find ways to supplement the training set so that the CNN can continue to learn phenological features. This can be achieved by pooling images from multiple public datasets, data augmentation techniques, or supplementation with images of related species (Christin et al., 2019). For this reason, future endeavors could evaluate the accuracy of CNNs trained on community science image sets that contain multiple species.

Since its inception in 2013, the iNaturalist platform has seen explosive growth in the number of images uploaded to the platform. More than 12 million research-grade observations were recorded in 2020, up from 7 million observations in 2019 and 3 million in 2018 (iNaturalist, 2019, 2020, 2021)^5,6,7^. These records are extensive and allow phenology researchers to study phenology across entire species ranges, climate regions, ecoregions, and more (Di Cecco et al., 2021). Community science datasets will become even more valuable to phenology researchers over time as new images come online and extend the temporal coverage of the data (Mayer, 2010). However, in order to take full advantage of community science datasets, new tools are needed to extract phenological data inexpensively and efficiently from images. We have shown that CNNs can successfully extract phenological data from iNaturalist image sets, with comparable accuracy to manual annotation by humans, but at a lower labor cost and higher speed. This tool offers a promising solution to integrate community science datasets with cutting-edge phenological research and expand the scope of this field.

## Data Availability Statement

The original contributions presented in the study are included in the article/Supplementary Material. All model code and training information have been made available in a Github repository (https://github.com/naziz4/DeepPhenology), as well as a DRYAD repository (https://doi.org/10.5061/dryad.mkkwh7123). Further inquiries can be directed to the corresponding author/s.

## Author Contributions

RR and SK developed the idea for this project. RR led the project design, data analysis, and writing. NA constructed the CNNs with the assistance of SL and JK. JH assisted with project design. All authors contributed to writing and editing of the manuscript.

## Conflict of Interest

The authors declare that the research was conducted in the absence of any commercial or financial relationships that could be construed as a potential conflict of interest.

## Publisher’s Note

All claims expressed in this article are solely those of the authors and do not necessarily represent those of their affiliated organizations, or those of the publisher, the editors and the reviewers. Any product that may be evaluated in this article, or claim that may be made by its manufacturer, is not guaranteed or endorsed by the publisher.

## Footnotes

1
https://www.inaturalist.org/stats/2020

2
https://ebird.org/news/2020-year-in-review

3
plantphenology.org

4
https://pytorch.org/tutorials/beginner/transfer_learning_tutorial.html

5
https://www.inaturalist.org/stats/2020

6
https://www.inaturalist.org/stats/2019

7
https://www.inaturalist.org/stats/2018
