# Supplementary material for: Using Convolutional Neural Networks to Efficiently Extract Immense Phenological Data From Community Science Images
Source: Front Plant Sci. 2022 Jan 17;12:787407. doi: 10.3389/fpls.2021.787407 (PMC8801702; doi:10.3389/fpls.2021.787407)
Supplement: Supplementary file 1 [file Table_1.docx]

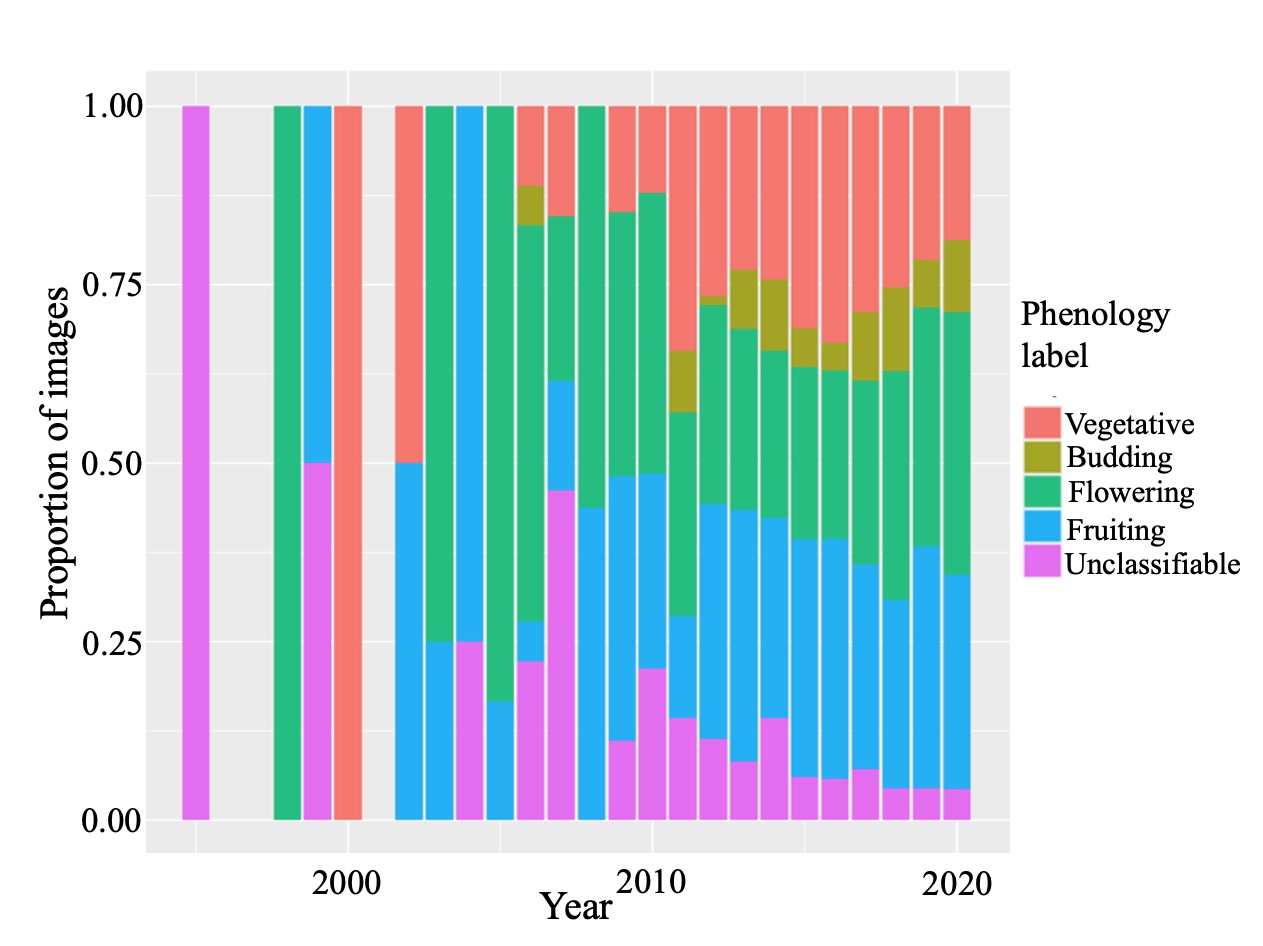


**Supplementary Figure 1.** Proportion of images in each four-stage phenology category, grouped by year. “Unclassifiable” images were unable to be labeled for phenology and were removed from the final four-stage training and validation dataset. All images (n = 12,758) were manually labeled by an experienced annotator.
